# CXCL9-Derived Peptides Differentially Inhibit Neutrophil Migration *In Vivo* through Interference with Glycosaminoglycan Interactions

**DOI:** 10.3389/fimmu.2017.00530

**Published:** 2017-05-10

**Authors:** Vincent Vanheule, Daiane Boff, Anneleen Mortier, Rik Janssens, Björn Petri, Elzbieta Kolaczkowska, Paul Kubes, Nele Berghmans, Sofie Struyf, Andreas J. Kungl, Mauro Martins Teixeira, Flavio Almeida Amaral, Paul Proost

**Affiliations:** ^1^Laboratory of Molecular Immunology, Department of Microbiology and Immunology, Rega Institute for Medical Research, KU Leuven, Leuven, Belgium; ^2^Departamento de Fisiologia e Biofisica, Instituto de Ciencias Biologicas, Universidade Federal de Minas Gerais, Belo Horizonte, Minas Gerais, Brazil; ^3^Mouse Phenomics Resource Laboratory, Department of Microbiology, Immunology and Infectious Diseases, Cumming School of Medicine, Snyder Institute for Chronic Diseases, University of Calgary, Calgary, AB, Canada; ^4^Department of Evolutionary Immunology, Institute of Zoology, Jagiellonian University, Krakow, Poland; ^5^Laboratory of Immunobiology, Department of Microbiology and Immunology, Rega Institute for Medical Research, KU Leuven, Leuven, Belgium; ^6^Immunology Research Group, Department of Physiology and Pharmacology, Snyder Institute for Chronic Diseases, University of Calgary, Calgary, AB, Canada; ^7^Department of Pharmaceutical Chemistry, Institute of Pharmaceutical Sciences, Karl-Franzens Universität, Graz, Austria

**Keywords:** chemokine, CXCL9, glycosaminoglycan, neutrophil, anti-inflammatory, gout, cremaster muscle

## Abstract

Several acute and chronic inflammatory diseases are driven by accumulation of activated leukocytes due to enhanced chemokine expression. In addition to specific G protein-coupled receptor-dependent signaling, chemokine–glycosaminoglycan (GAG) interactions are important for chemokine activity *in vivo*. Therefore, the GAG–chemokine interaction has been explored as target for inhibition of chemokine activity. It was demonstrated that CXCL9(74-103) binds with high affinity to GAGs, competed with active chemokines for GAG binding and thereby inhibited CXCL8- and monosodium urate (MSU) crystal-induced neutrophil migration to joints. To evaluate the affinity and specificity of the COOH-terminal part of CXCL9 toward different GAGs in detail, we chemically synthesized several COOH-terminal CXCL9 peptides including the shorter CXCL9(74-93). Compared to CXCL9(74-103), CXCL9(74-93) showed equally high affinity for heparin and heparan sulfate (HS), but lower affinity for binding to chondroitin sulfate (CS) and cellular GAGs. Correspondingly, both peptides competed with equal efficiency for CXCL8 binding to heparin and HS but not to cellular GAGs. In addition, differences in anti-inflammatory activity between both peptides were detected *in vivo*. CXCL8-induced neutrophil migration to the peritoneal cavity and to the knee joint were inhibited with similar potency by intravenous or intraperitoneal injection of CXCL9(74-103) or CXCL9(74-93), but not by CXCL9(86-103). In contrast, neutrophil extravasation in the MSU crystal-induced gout model, in which multiple chemoattractants are induced, was not affected by CXCL9(74-93). This could be explained by (1) the lower affinity of CXCL9(74-93) for CS, the most abundant GAG in joints, and (2) by reduced competition with GAG binding of CXCL1, the most abundant ELR^+^ CXC chemokine in this gout model. Mechanistically we showed by intravital microscopy that fluorescent CXCL9(74-103) coats the vessel wall *in vivo* and that CXCL9(74-103) inhibits CXCL8-induced adhesion of neutrophils to the vessel wall in the murine cremaster muscle model. Thus, both affinity and specificity of chemokines and the peptides for different GAGs and the presence of specific GAGs in different tissues will determine whether competition can occur. In summary, both CXCL9 peptides inhibited neutrophil migration *in vivo* through interference with GAG interactions in several animal models. Shortening CXCL9(74-103) from the COOH-terminus limited its GAG-binding spectrum.

## Introduction

Several acute and chronic inflammatory diseases, such as gout and rheumatoid arthritis, are characterized by enhanced expression of chemokines and accumulation of activated leukocytes in tissues (1–6). Chemokines or *chem*otactic cyto*kines* are proteins, consisting of 60–110 amino acids, which play an important role in the migration of leukocytes (7–10). Chemokines are classified into C, CC, CXC, and CX_3_C subfamilies, based on the arrangement of conserved NH_2_-terminal cysteine motifs. For most chemokines an alternative biological classification can be made between homeostatic or constitutively expressed chemokines, and inflammatory or inducible chemokines. The latter subclass is locally secreted by tissue cells and resident leukocytes upon infection or tissue damage, thereby creating a gradient along which leukocytes can migrate from the blood vessel to the site of inflammation (11–14). To create such a leukocyte migration inducing gradient, it is necessary that chemokines are presented on the endothelium at the site of inflammation through binding to glycosaminoglycans (GAGs), thereby preventing chemokine diffusion and degradation and retaining high local chemokine concentrations. Subsequently, GAG-bound chemokines interact with their G protein-coupled receptors (GPCRs), expressed by specific circulating leukocyte subtypes. This results in adhesion to and extravasation of leukocytes through the endothelium (15–17). Once leukocytes enter the tissue, they can migrate to the site of inflammation through the gradient of local GAG-bound chemokines. The binding of chemokines to GAGs has been proven to be indispensable for chemokine activity *in vivo* (18–20). Chemokine variants with mutations in their GAG-binding motif showed inactive *in vivo* due to abrogated GAG binding, although receptor binding and chemotactic activity *in vitro* are seldom affected (21, 22). In addition, leukocytes of mice with disturbed heparan sulfate (HS) synthesis in endothelial cells and leukocytes showed reduced chemokine-induced migration (23).

Because of their essential role for the migration of leukocytes, chemokines and their GPCRs can serve as potential targets for the development of new anti-inflammatory drugs (24–26). Several neutralizing ligands or antibodies, modified chemokines or small molecules have been developed as antagonists (27–30). However, only two chemokine receptor antagonists are currently used as therapeutics, namely, Maraviroc (a CCR5 antagonist) and AMD3100 (a CXCR4 antagonist) (31, 32). Remarkably, these antagonists are not used as anti-inflammatory drugs, but, respectively, as an inhibitor of human immunodeficiency virus infection and as a stem cell mobilizer. Recently, intact modified chemokines were developed that interfere with the binding of chemokines to GAGs (33, 34). These modified chemokines have, in comparison with their natural human chemokine counterpart, an enhanced affinity for GAGs but a decreased affinity for their GPCRs. In this way, the modified chemokines or decoy chemokines can compete with functional chemokines for GAG binding. Thereby, they reduce chemokine immobilization and presentation and enhance the inhibition of chemokine-induced leukocyte migration. It has been shown that PA401, a CXCL8-based decoy protein, exerts strong anti-inflammatory activity *in vivo* (35–38). Also CCL2- and CXCL12-based decoy proteins with high GAG-binding affinity and reduced activity on GPCRs have been developed (39, 40). Interference with GAG interactions also forms the basis for the inhibition of lymphocyte migration with the spiegelmer NOX-A12 (41). This RNA oligonucleotide competes with GAGs for the binding to CXCL12, leading to neutralization of CXCL12 activity *in vivo*. More recently, COOH-terminal peptides of the chemokine CXCL9 or monokine induced by interferon-γ (MIG) were described as potent GAG-binding peptides (42, 43). The CXCL9(74-103) peptide could inhibit the *in vivo* chemotactic activity of CXCL8 by competing with CXCL8 for the binding to GAGs. Also in a murine model of monosodium urate (MSU) crystal-induced gout, the peptide was able to inhibit neutrophil extravasation. Reducing the length of the CXCL9 peptide gradually decreased the capacity to compete with CXCL8 for GAG binding and CXCL9(86-103) was unable to inhibit chemotactic activity of CXCL8 *in vivo*. These data stress the importance of amino acids 74–78 and two typical heparin-binding motifs (BBXB and BBBXXB), present in CXCL9(74-103), for the GAG-binding characteristics.

In this study, we investigated in more detail the specificity of chemokine-derived GAG-binding peptides and we evaluated the potential of such peptides to interfere with *in vivo* neutrophil adhesion and migration to different tissues (peritoneal cavity, joints, and muscles) in mice.

## Materials and Methods

### Cells

Chinese hamster ovary (CHO) cells were a gift from M. Parmentier (ULB, Brussels, Belgium) and were cultured in Ham’s F-12 medium (with l-glutamine; Lonza, Belgium) supplemented with 10% fetal calf serum (FCS) and 0.8% G418 (Geneticin^®^; Life Technologies), as described (44). To inhibit sulfation of cellular GAGs, CHO cells were cultured in the presence of 100 mM sodium chlorate (NaClO_3_) for 24 h as previously described (42).

### Mice

Animal experiments at the Rega Institute for Medical Research, University of Leuven, were carried out in female adult NMRI mice, purchased from Elevage Janvier (Le Genest Saint Isle, France), in agreement with the Ethical Committee for Animal Care and Use of the KU Leuven and in adherence to the international guidelines for animal ethics and welfare. Mouse studies at the Federal University of Minas Gerais were performed in adult C57BL/6J mice. The experiments were approved by the Animal Ethical Committee of the Federal University of Minas Gerais. Mice were sacrificed by i.p. injection of an overdose of nembutal or ketamine (150 mg/kg) and xylazine (20 mg/kg) prior to the collection of joint lavages. For the intravital microscopy studies, male C57Bl/6J mice were purchased from Jackson Laboratories (Bar Harbor, ME, USA). Animals were maintained in a specific pathogen-free environment at the University of Calgary Animal Resource Centre. All experimental animal protocols were approved by the University of Calgary Animal Care Committee and were in compliance with the Canadian Council for Animal Care Guidelines.

### Solid-Phase Synthesis of COOH-Terminal Peptides

COOH-terminal peptides of CXCL4 [CXCL4(47-70)] and CXCL9 [CXCL9(74-103), CXCL9(74-93), CXCL9(86-103)] were chemically synthesized using fluorenyl methoxycarbonyl (Fmoc) chemistry using an Activo-P11 automated synthesizer (Activotec, Cambridge, UK), as previously described (45). Part of the material was site-specifically biotinylated or fluorescently labeled at the NH_2_-terminus using biotin-p-nitrophenyl ester (Novabiochem, Darmstadt, Germany) or 5(6)-carboxytetramethylrhodamine (TAMRA; Merck Millipore, Darmstadt, Germany), respectively (42). After synthesis, intact synthetic peptides were purified and identified by mass spectrometry (Amazon SL or Amazon Speed ETD ion trap, Bruker, Bremen, Germany).

### Isothermal Fluorescence Titration (IFT)

To study the interaction between CXCL9(74-93) and GAGs, titration experiments were performed as described by Gerlza et al. (46) using TAMRA-labeled synthetic CXCL9(74-93), TAMRA-labeled synthetic CXCL9(74-103), HS (Iduron BN1, Manchester, UK), low molecular weight heparin (LMWH; Iduron BN5), and chondroitin sulfate (CS; Carbosynth, Compton, UK).

### Binding of COOH-Terminal Peptides to Cellular GAGs and Competition with CXCL8

Binding of biotinylated CXCL9(74-103) and CXCL9(74-93) to CHO cells was assessed by flow cytometry as recently described (42). Briefly, cells were detached with phosphate-buffered saline (PBS) with 0.02% ethylenediaminetetraacetic acid (EDTA) and incubated with NH_2_-terminally biotinylated peptides. Subsequently, cells were incubated with streptavidin-allophycocyanin (BD Biosciences, San Jose, CA, USA) and analyzed by flow cytometry (FACSCalibur, BD Biosciences). To confirm inhibition of sulfation of cellular GAGs, part of the cells were stained with a mouse monoclonal anti-human HS antibody (Immunosource, Schilde, Belgium) and a secondary phycoerythrin-labeled goat anti-mouse antibody (BD Biosciences). Finally, cells were analyzed by flow cytometry. Analogously, binding of 100 nM biotinylated CXCL8 in the presence or absence of CXCL9(74-103) or CXCL9(74-93) was compared on CHO cells.

### Competition of COOH-Terminal Peptides for Binding of Chemokines to GAGs

The ability of the COOH-terminal peptides of CXCL9 and CXCL4 to compete for GAG binding with the inflammatory chemokines CXCL8 and CXCL1 was evaluated on heparin-binding plates [BD Biosciences or produced by Jason Whittle (School of Engineering/Future Industries Institute, University of South Australia, Australia)] (42, 47). In brief, GAGs (heparin, HS, and CS; Iduron; 25 µg/ml) were coated overnight at room temperature. Dilutions of COOH-terminal CXCL9 or CXCL4 peptide combined with recombinant human CXCL8(1-77) or recombinant murine CXCL1(1-72) (Peprotech, Rocky Hill, NJ, USA) were added in duplicate and incubated for 2 h at 37°C. Subsequently, bound CXCL8 or CXCL1 was detected with biotinylated polyclonal rabbit anti-human CXCL8 or biotinylated polyclonal goat anti-mouse CXCL1 (Peprotech) and horse radish peroxidase-labeled streptavidin. Finally, the peroxidase activity was quantified at 450 nm.

### CXCL8-Induced Migration of Neutrophils *In Vivo*

*In vivo* neutrophil extravasation to the peritoneal or the knee cavity was examined, respectively, by intraperitoneal or intra-articular injection, of endotoxin-free recombinant CXCL8(1-77) (PeproTech) or vehicle (PBS) in female NMRI or C57BL/6 mice, as described previously (42, 48). Simultaneously, synthetic CXCL9 peptide was co-injected either intraperitoneally or injected intravenously. After 2 h (peritoneal cavity) or 3 h (knee cavity), mice were sacrificed. The peritoneal cavity was washed with saline enriched with 2% FCS and 20 U/ml heparin and total leukocyte counts were determined. For the preparation of cytospins, 10^5^ cells were used and absolute number and percentage of neutrophils in the lavages were determined in duplicate independently by two researchers by differential microscopic counting. The cells present in the tibiofemoral articulation were harvested by washing the cavity with 10 µl of 3% (w/v) bovine serum albumin in PBS and diluted in 90 µl of 3% (w/v) bovine serum albumin in PBS. The number of total leukocytes was determined by counting leukocytes in a Neubauer chamber after staining with Turk’s solution. Differential counts were obtained from cytospin preparations by evaluating the percentage of each leukocyte type on a slide stained with May-Grunwald-Giemsa solution.

Intravenously injected fluorescent CXCL9(74-103) was detected in plasma after different time intervals by spectrofluorometric analysis and quantified against murine plasma spiked with known concentrations of the fluorescent peptide.

### MSU Crystal-Induced Gout in Mice

Monosodium urate crystals were prepared as described previously (42, 49) and were injected (100 µg in 10 µl) in the tibiofemoral knee joint of C57Bl/6 mice. A group of mice received CXCL9 peptide or vehicle intravenously and the cells present in the tibiofemoral articulation were harvested 3 h after MSU crystal administration. The total and differential leukocyte counts were determined.

### *In Vivo* Neutrophil Recruitment into the Cremaster Muscle

Mice were anesthetized by intraperitoneal injection of 200 mg/kg ketamine (Bayer Inc. Animal Health) and 10 mg/kg xylazine (Bimeda-MTC). After anesthesia, the right jugular vein was cannulated for maintenance of anesthesia and to permit the delivery of the fluorescence-labeled antibodies. Mice were injected intravenously (via jugular vein) with either saline or CXCL9(74-103) 5 min prior to an intrascrotal injection of 1 or 3 µg CXCL8. The animals were placed on a water-heated operating table to maintain body temperature at ~37°C. The depth of anesthesia was controlled by regularly monitoring peripheral reflexes. The cremaster muscle was prepared as previously described (50). Briefly, the muscle was dissected free from other tissues and opened longitudinally with cautery. The muscle was held flat on a cover slip by attaching five sutures in the corner of the tissue and the tissue was then constantly superperfused (1 ml/min) with pre-warmed bicarbonate buffered saline (pH 7.4), throughout the experiment. Neutrophils were stained by intravenous injected anti-Ly6G-A647 home labeled antibodies (anti-Ly6G, clone 1A8, was purchased from BioLegend and coupled to Alexa647 with the ThermoFisher protein labeling kit). In experiments in which unlabeled CXCL9(74-103) was used (evaluation of neutrophil adherence), neutrophils were labeled with PE-conjugated anti-Ly6G antibodies (eBiosciences) and endothelium with anti-CD31-A647 antibodies (BD Biosciences and coupled to Alexa647 with the ThermoFisher protein labeling kit). Single unbranched cremasteric venules with a diameter of ~25–40 μm were selected and their blood flow was recorded during a 5 min period. Spinning disk confocal intravital microscopy was performed using an Olympus BX51W1 (Olympus, Center Valley, PA, USA) upright microscope equipped with a 20×/0.95 XLUM Plan Fl water immersion objective. The microscope was equipped with a confocal light path (WaveFx, Quorum, Guelph, ON, Canada) on a Yokogawa CSU-10 head (Yokogawa Electric Corporation, Tokyo, Japan). Sensitivity settings were maintained at the same level for all experiments. A 512 × 512 pixel back-thinned EMCCD camera (C9100-13, Hamamatsu, Bridgewater, NJ, USA) was used for fluorescence detection. Volocity Acquisition software (Improvision Inc., Lexington, MA, USA) was used to drive the confocal microscope. Images captured using the spinning disk were processed and analyzed in Volocity 4.20. Periods of 5 min/field of view were recorded at 45, 60, and 90 min after CXCL8 administration. A neutrophil was considered to be adherent if it remained stationary for at least 30 s, and total neutrophil adhesion was quantified as the number of adherent cells within a 100-µm length of venule.

Alternatively, to evaluate the stability of the peptide on vascular endothelium, fluorescent CXCL9(74-103) was injected intravenously and visualized by intravital microscopy after different time intervals.

### Statistical Analysis

All statistical analyses were performed using Statistica software version 13.1 (Dell, USA). The Mann–Whitney *U*-test was used to compare means or medians from independent groups (*p < 0.05; **p < 0.01; ***p < 0.005).

## Results

### Chemical Synthesis and Labeling of CXCL9(74-103) and CXCL9(74-93)

**Figure 1:**
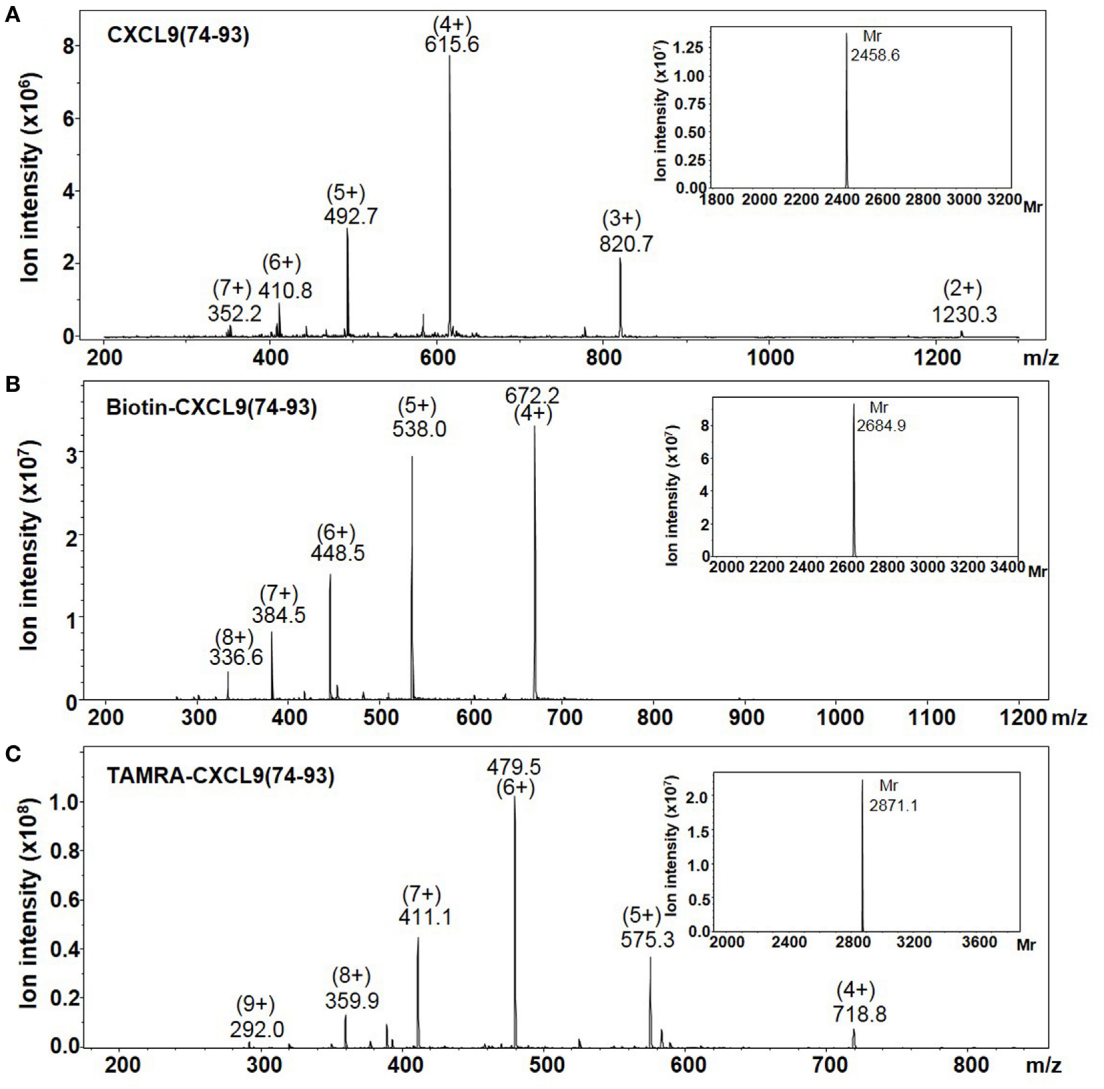
**The averaged mass spectra of CXCL9(74-93) peptides**. The COOH-terminal peptides CXCL9(74-93), NH_2_-terminally biotinylated CXCL9(74-93), and NH_2_-terminally TAMRA-labeled CXCL9(74-93) were chemically synthesized based on Fmoc-chemistry. The intensity of the detected ions in function of their specific mass/charge (*m*/*z*) ratio is shown for purified CXCL9(74-93) **(A)**, NH_2_-terminally biotinylated CXCL9(74-93) **(B)**, and NH_2_-terminally biotinylated TAMRA-labeled CXCL9(74-93) **(C)**. In order to calculate the relative molecular mass (*M*_r_) of the proteins corresponding to the indicated ions, Bruker deconvolution software was used. The experimentally determined *M*_r_ of the peptides are shown as inserts on the upper right of the averaged mass spectra.

The highly cationic COOH-terminal peptides of CXCL9, namely, CXCL9(86-103), CXCL9(74-103), and CXCL9(74-93), were chemically synthesized, deprotected, and purified by RP-HPLC to evaluate their biological function (Table 1). Part of the peptides was site-specifically biotinylated or fluorescently labeled at the NH_2_-terminus. The *M*_r_ of the unlabeled and labeled purified synthetic peptides was confirmed by ion trap mass spectrometry. Figure 1 shows the mass spectra of CXCL9(74-93), NH_2_-terminally biotinylated CXCL9(74-93), and NH_2_-terminally TAMRA-labeled CXCL9(74-93). The experimentally determined *M*_r_ corresponded to the theoretical *M*_r_ of 2,460.1, 2,686.4, and 2,871.5 for CXCL9(74-93), biotin-CXCL9(74-93), and TAMRA-CXCL9(74-93), respectively. In addition, a COOH-terminal peptide of CXCL4, namely, CXCL4(47-70), was chemically synthesized and purified (51).

**Table 1:** **Overview of the amino acid sequence of the COOH-terminal peptides of CXCL9 and CXCL4 synthetized to investigate their glycosaminoglycan-binding affinity and their anti-inflammatory function**.

Peptide	Amino acid sequence	*M*_r_ (Da)
CXCL9(74-103)	_74_KKKQKNGKKHQKKKVLKVRKSQRSRQKKTT_103_	3,661.4
CXCL9(74-93)	_74_KKKQKNGKKHQKKKVLKVRK_93_	2,460.1
CXCL9(86-103)	_86_KKVLKVRKSQRSRQKKTT_103_	2,199.7
CXCL4(47-70)	_47_NGRKICLDLQAPLYKKIIKKLLES_70_	2,785.4
Biotin-CXCL9(74-93)	Biotin-_74_KKKQKNGKKHQKKKVLKVRK_93_	2,686.4
TAMRA-CXCL9(74-93)	TAMRA-_74_KKKQKNGKKHQKKKVLKVRK_93_	2,871.5
Biotin-CXCL9(74-103)	Biotin-_74_KKKQKNGKKHQKKKVLKVRKSQRSRQKKTT_103_	3,887.7
TAMRA-CXCL9(74-103)	TAMRA-_74_KKKQKNGKKHQKKKVLKVRKSQRSRQKKTT_103_	4,072.8

*The amino acid sequence and the M_r_ are listed. For clarity, basic amino acids were underlined*.

### Interaction of CXCL9(74-93) with Soluble and Cellular GAGs

To obtain more insight in the contribution of the unique COOH-terminal region of CXCL9 and in the specificity of CXCL9-derived peptides for different GAGs, the affinity of CXCL9(74–93) for LMWH, HS, and CS was determined by IFT. The affinity (*K*_D_ value) of CXCL9(74-93) for LMWH, namely, 63 ± 13 nM, was comparable to the previously reported *K*_D_ for LMWH of the longer CXCL9(74-103) peptide (61 nM) (42). A biphasic binding isotherm was obtained when titrating CXCL9(74-93) with HS, indicating more than one binding site for the peptide on this GAG. Fitting only the initial high-affinity phase, a *K*_D_ value of 1 nM for HS was obtained. In contrast to the comparable *K*_D_ values on LMWH, CXCL9(74-103) showed about fourfold higher affinity for CS (68 ± 4.6 nM) compared to CXCL9(74-93) (296 ± 19 nM).

**Figure 2:**
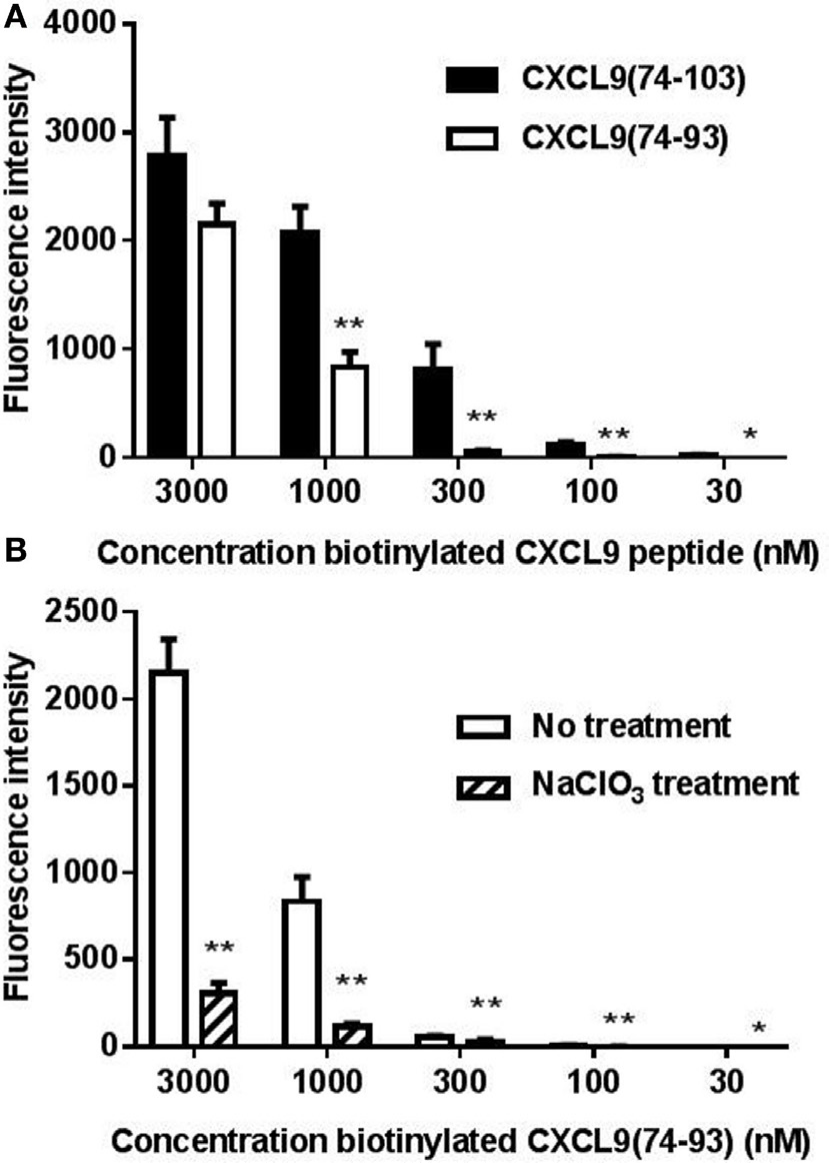
**CXCL9(74-93) interacts with cellular glycosaminoglycans (GAGs)**. The interaction of the COOH-terminal CXCL9 peptides with Chinese hamster ovary (CHO) cells was assessed by flow cytometric analysis. **(A)** CHO cells were treated with dilutions of NH_2_-terminally biotinylated CXCL9(74-103) and CXCL9(74-93), which were detected by streptavidin-allophycocyanin. **(B)** To ensure that the binding of CXCL9(74-93) was GAG-mediated, CHO cells were treated with sodium chlorate (NaClO_3_) to reduce the sulfation of GAGs. Binding of biotinylated CXCL9(74-93) to cells, treated or untreated with NaClO_3_, was detected by streptavidin-allophycocyanin. The mean (+SEM) fluorescence intensity of bound biotinylated CXCL9 peptides to CHO cells is indicated on the *y*-axis (*n* ≥ 4). A statistical comparison to evaluate the binding of biotinylated peptides on CHO cells [compared to the corresponding concentration of CXCL9(74-103) **(A)** or to the binding to cells not treated with NaClO_3_ **(B)**] was performed using the Mann–Whitney *U*-test (*p < 0.05; **p < 0.01).

Because binding of the peptides to cellular GAGs is physiologically more relevant, binding of biotinylated CXCL9(74-103) and CXCL9(74-93) to CHO cells was evaluated by flow cytometric analysis. Figure 2A shows dose-dependent binding of the CXCL9 peptides to CHO cells. Although CXCL9(74-93) and CXCL9(74-103) bound with comparable affinity to LMWH, CXCL9(74-93) displayed lower affinity to cellular GAGs. To ensure that binding was GAG-mediated, CHO cells were treated with NaClO_3_ to reduce the sulfation of GAGs. Measuring HS expression with a specific anti-HS antibody provides an indication for the total GAG expression level, as HS is the most relevant and abundant GAG on CHO and endothelial cells (52). Treatment of the CHO cells with 100 mM NaClO_3_ significantly reduced the expression of HS with 71.4% as evaluated by flow cytometry (data not shown) and consequently the binding of CXCL9(74-93) (Figure 2B).

### CXCL9(74-93) Competes with CXCL8 for Binding to Heparin or Cellular GAGs

**Figure 3:**
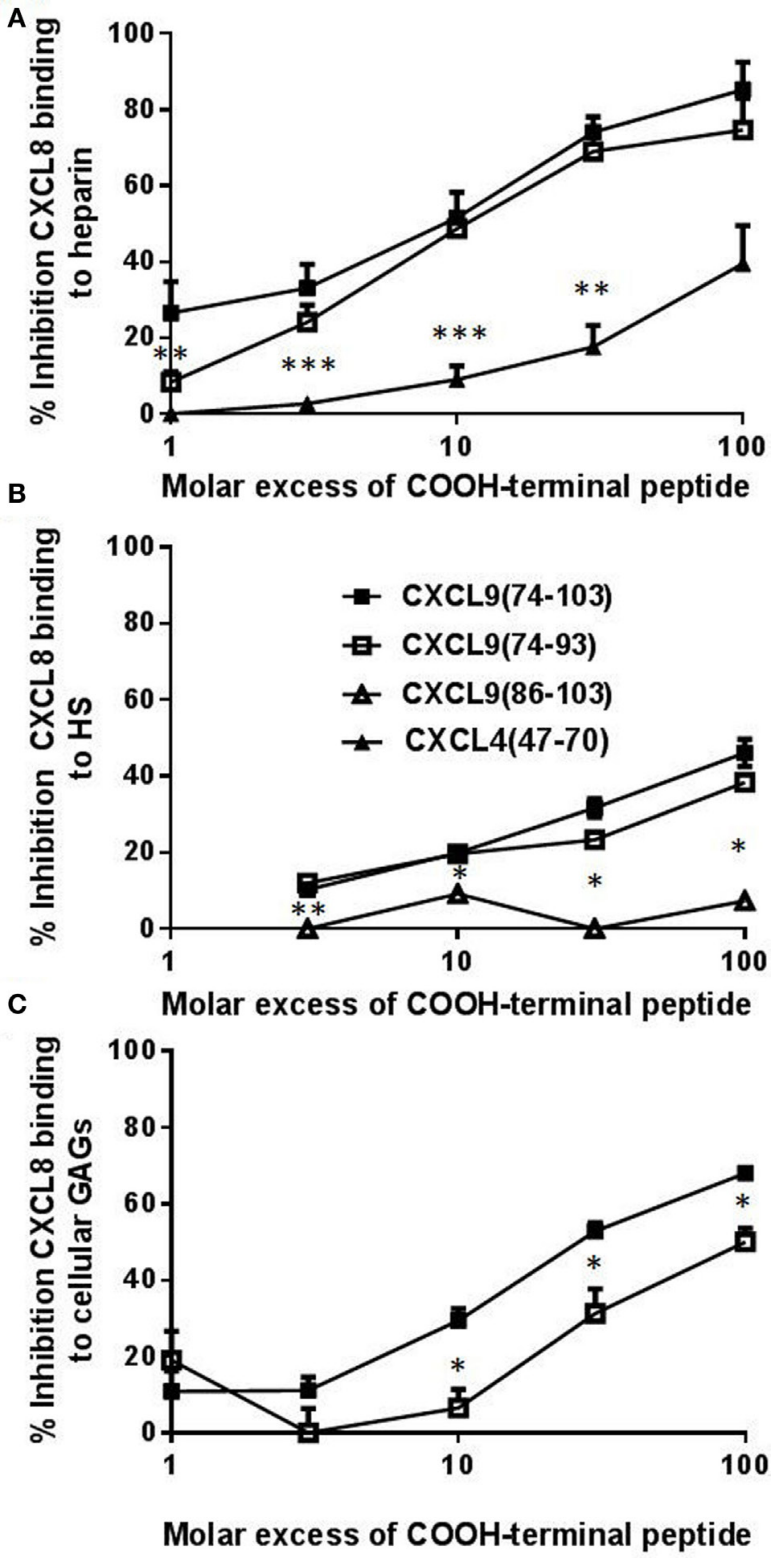
**CXCL9(74-93) competes with CXCL8 for binding to heparin, heparan sulfate (HS), and cellular glycosaminoglycans (GAGs)**. Competition between CXCL8 and COOH-terminal CXCL9 or CXCL4 peptides for binding to immobilized heparin **(A)** or HS **(B)** and to Chinese hamster ovary (CHO) cells **(C)** was performed. **(A)** and **(B)** CXCL8(1-77) (0–300 nM) was added to heparin-coated or HS-coated 96-well plates in the presence or absence of the indicated excess of COOH-terminal peptide [CXCL9(74-103) (■), CXCL9(74-93) (□), CXCL9(86-103) (Δ), CXCL4(47-70) (▴)]. Bound CXCL8 was detected with biotinylated anti-human CXCL8 antibodies. **(C)** Binding of NH_2_-terminally biotinylated CXCL8(1-77) (300 nM) to CHO cells in the presence or absence of an excess CXCL9 peptide was detected by streptavidin-allophycocyanin and flow cytometry. The mean (± SEM) percentage inhibition of binding of CXCL8(1-77) to heparin, HS or cellular GAGs is indicated on the *y*-axis (*n* ≥ 4). Statistical comparison of the different competitors with CXCL9(74–103) was performed using the Mann–Whitney *U-*test (*p < 0.05).

As recently described, CXCL9(74-103) more potently competes with CXCL8 for binding to heparin than CXCL9(74-103) missing 5 to 12 NH_2_-terminal residues, stressing the importance of amino acids 74–78 for heparin binding (42). To further investigate the role of the COOH-terminal amino acids, the ability of CXCL9(74-93) to inhibit binding of chemokines to heparin and HS was evaluated. CXCL9(74-93) and CXCL9(74-103) together with a COOH-terminal peptide of one of the strongest GAG-binding chemokines, namely, CXCL4(47-70), were compared in an ELISA-like assay to compete with CXCL8 for heparin binding. As evidenced in Figure 3, CXCL9(74-93) competed with CXCL8 for heparin binding with a similar potency as CXCL9(74-103) thereby stressing the importance of amino acids 74–93. For both COOH-terminal CXCL9 peptides, a 10-fold molar excess compared to CXCL8 resulted in a 50% reduction of CXCL8 binding to heparin (Figure 3A). Recently, it has been shown that CXCL4(47-70) binds dose-dependently to GAGs on CHO cells with about fivefold lower affinity than CXCL9(74-103) (42). Indeed, also in the ELISA-like heparin-binding assay, CXCL4(47-70) was a less potent competitor of CXCL8 binding than the CXCL9 peptides. A more than 100-fold molar excess of CXCL4(47-70) was needed to achieve a 50% reduction of CXCL8 binding. On HS-coated plates, CXCL9(74-93) and CXCL9(74-103) were equally efficient in inhibiting CXCL8 binding (Figure 3B), in contrast to the poorly competing CXCL9(86-103). Also on CHO cells, CXCL9(74-93) could inhibit the binding of biotinylated CXCL8 to cellular GAGs by 31% in the presence of a 30-fold excess of CXCL9(74-93) (Figure 3C). A 100-fold excess of CXCL9(74-93) resulted in 50% inhibition. On the other hand, a 30-fold excess of CXCL9(74-103) could inhibit the binding of CXCL8 by 53%.

### CXCL9 Peptides Inhibit CXCL8- and MSU Crystal-Induced Neutrophil Recruitment *In Vivo*

**Figure 4:**
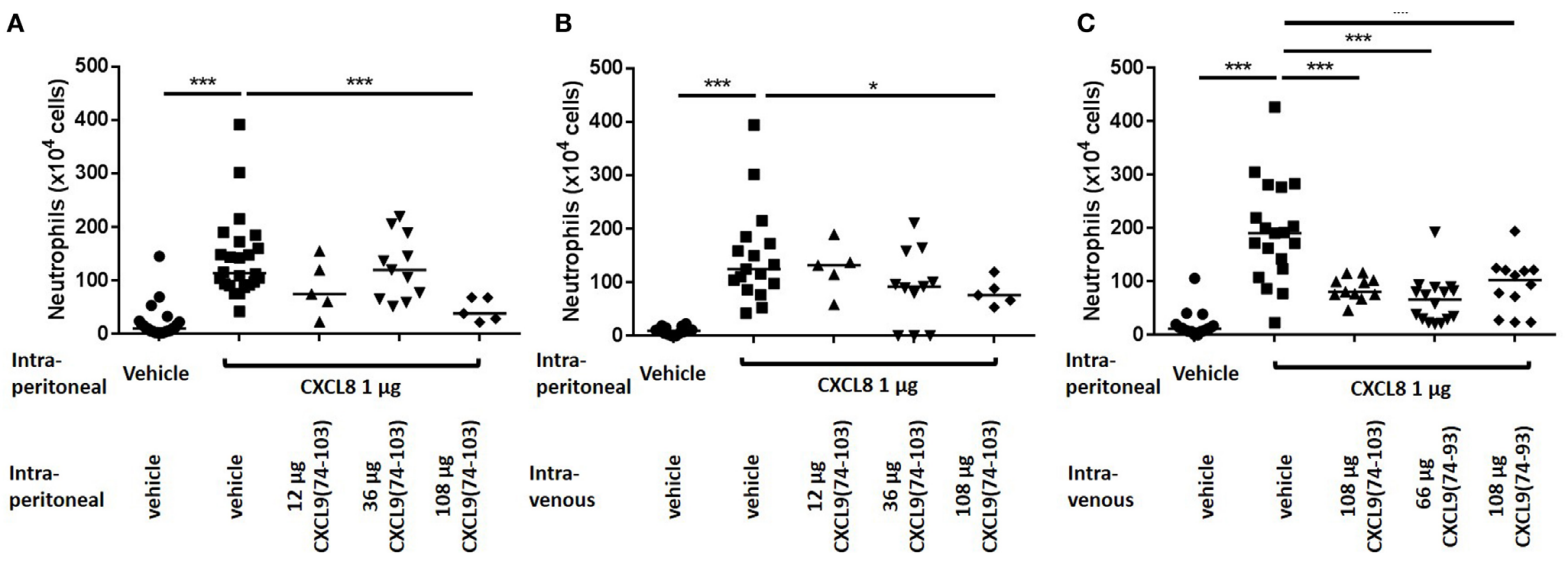
**The COOH-terminal peptides of CXCL9 inhibit CXCL8-induced neutrophil migration to the peritoneal cavity**. CXCL8(1-77) was injected into the peritoneal cavity of NMRI mice (*n* ≥ 5) simultaneously with an intraperitoneal **(A)** or intravenous **(B,C)** injection of CXCL9(74-103) or CXCL9(74–93). The number of neutrophils in the peritoneal cavity, 2 h post injection, were evaluated by differentially counting the leukocyte subtypes on Hemacolor-stained cytospins. The neutrophil counts are shown for every individual mouse, and the horizontal lines denote the median percentage. To detect statistically significant differences, the Mann–Whitney *U-*test was carried out (*p < 0.05; **p < 0.01; ***p < 0.005).

**Figure 5:**
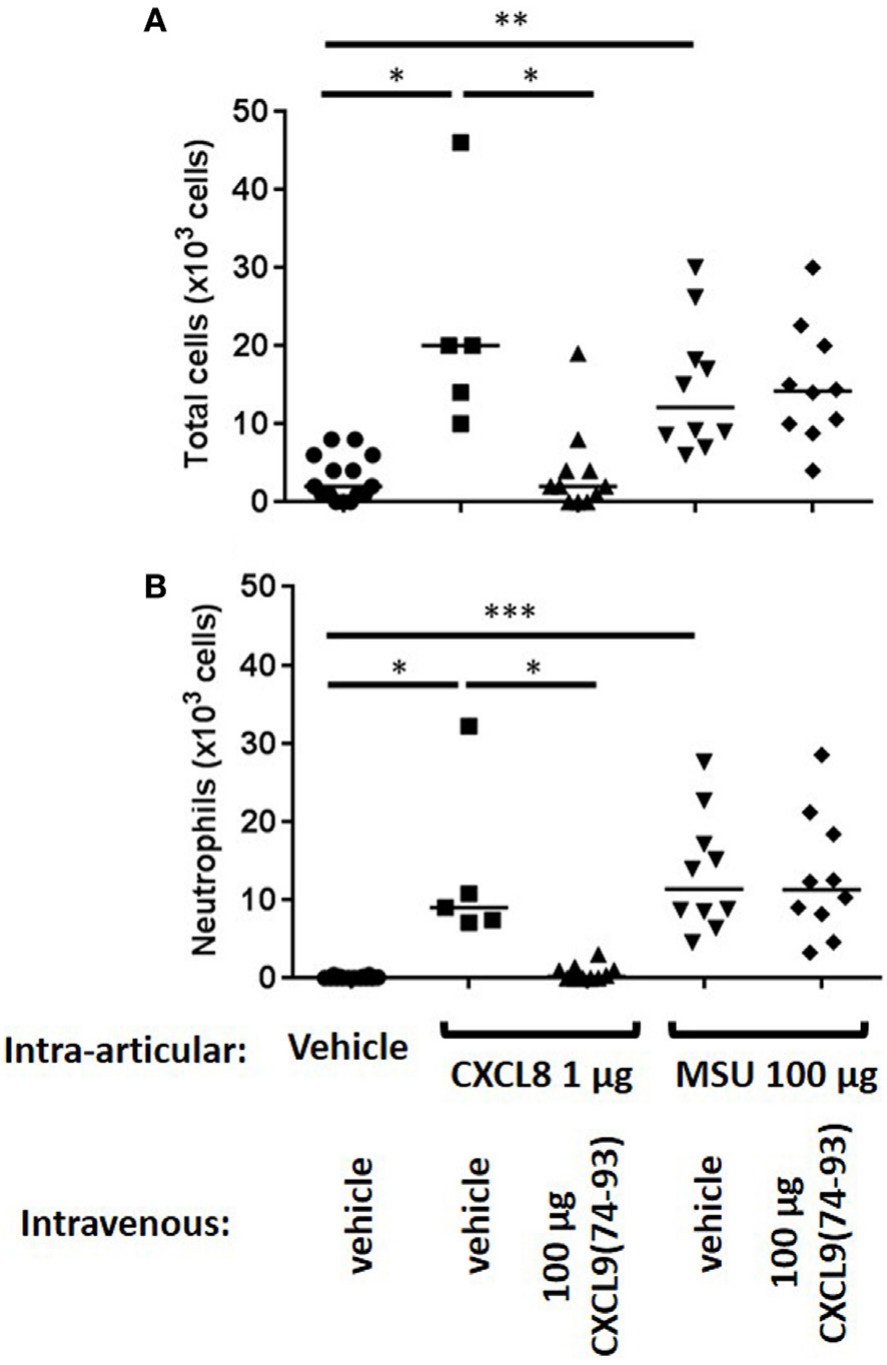
**CXCL9(74-93) inhibits CXCL8-induced but not monosodium urate (MSU) crystal-induced neutrophil migration to the knee cavity**. CXCL8(1-77) (1 µg) or MSU crystals (100 µg) were injected into the tibiofemoral articulation of C57BL/6 mice (*n* ≥ 5). Simultaneously, 66 µg of CXCL9(74-93) was injected intravenously and 3 h post injection the number of total cells **(A)** and neutrophils **(B)** were evaluated by washing the cavity and differentially counting the leukocyte subtypes on May-Grunwald-Giemsa-stained cytospins. The total numbers of leukocytes and neutrophils are shown for every individual mouse, and the horizontal lines denote the median number of cells. To detect statistically significant differences, the Mann–Whitney *U*-test was carried out (*p < 0.05; **p < 0.01; ***p < 0.005).

Recently, it was shown that CXCL9(74-103) could inhibit the neutrophil chemotactic activity of CXCL8 *in vivo* when CXCL8 was injected into the tibiofemoral articulation of C57BL/6 mice (42). To confirm this inhibition of CXCL8-induced neutrophil recruitment by CXCL9(74-103) in another body compartment, 1 µg of CXCL8 was injected intraperitoneally in NMRI mice. Injection of CXCL8 (1 µg) resulted in significant neutrophil migration to the peritoneal cavity, raising the absolute number and percentage of neutrophils in the peritoneal cavity to about 142 × 10^4^ neutrophils (±15%) compared to the saline-injected group (21 × 10^4^ neutrophils). Concomitant intraperitoneal injection of 108 µg CXCL9(74-103) significantly reduced the potency of CXCL8 to induce neutrophil extravasation (Figure 4A). Indeed, upon co-injection of 108 µg CXCL9(74-103), the neutrophil accumulation only increased to 45 × 10^4^ neutrophils (or 6.6%). In contrast, at lower doses tested, i.e., 12 and 36 µg, CXCL9(74-103) failed to reduce CXCL8-induced neutrophil recruitment. As it could be expected based on the heparin-binding assays conducted before (42), 108 µg intraperitoneally injected CXCL9(86–103) did not inhibit CXCL8-induced neutrophil migration (data not shown). Also intravenous injection of CXCL9(74-103) significantly reduced neutrophil migration toward intraperitoneally injected CXCL8 from 148 × 10^4^ neutrophils (16.5%) to 81 × 10^4^ neutrophils (10.1%) (Figure 4B). The lower doses of CXCL9(74-103) tested were not able to reduce the neutrophil migration significantly, although a trend toward a lower neutrophil influx was observed upon concomitant intravenous injection of 36 µg CXCL9(74-103). In addition, the inhibitory potential of CXCL9(74–93) was also evaluated in this *in vivo* model. Figure 4C shows that CXCL9(74–93) is an equally potent inhibitor of CXCL8 than CXCL9(74-103). Intravenous injection of 66 µg CXCL9(74-93), an equal molar amount compared to 108 µg CXCL9(74–103), significantly reduced the CXCL8-induced neutrophil migration to the peritoneum from 192 × 10^4^ neutrophils (19%) to 65 × 10^4^ neutrophils (8.25%). Also neutrophil extravasation to the joint was strongly reduced after intravenous injection of 66 µg CXCL9(74–93) from 13.3 × 10^3^ neutrophils (60.5%) to 0.67 × 10^3^ neutrophils (17.5%) (Figure 5).

**Figure 6:**
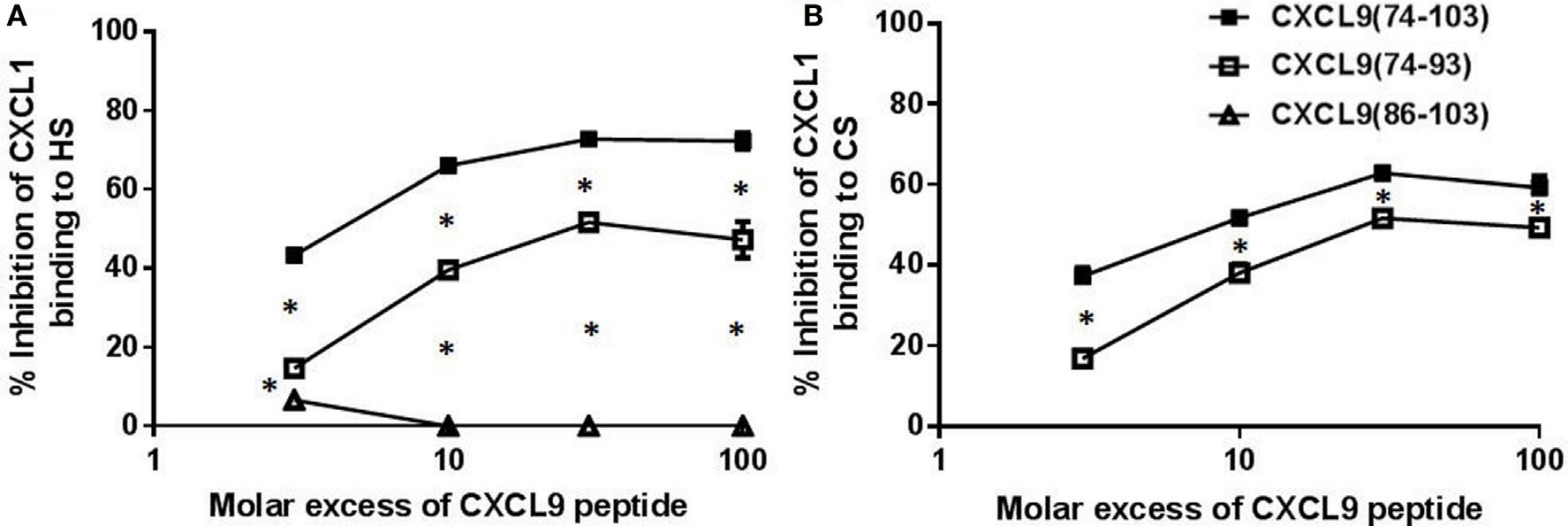
**CXCL9(74-93) is a less potent competitor for CXCL1 binding to glycosaminoglycans (GAGs)**. Competition between CXCL1 and COOH-terminal CXCL9 peptides for binding to immobilized heparan sulfate (HS) **(A)** and chondroitin sulfate (CS) **(B)** was performed. Dilutions of CXCL1 were added to GAGs in 96-well plates in the presence or absence of the indicated excess of COOH-terminal peptide [CXCL9(74-103) (■), CXCL9(74-93) (□), CXCL9(86-103) (Δ)]. Bound CXCL1 was detected with biotinylated polyclonal goat anti-mouse CXCL1 antibodies. The mean (± SEM) percentage inhibition of CXCL1 binding to GAGs is indicated on the *y-*axis (*n* ≥ 3). Statistical comparison of the different competitors with CXCL9(74–103) was performed using the Mann–Whitney *U-*test.

Interestingly and in contrast to CXCL9(74-103) (42), CXCL9(74-93) could not inhibit MSU crystal-induced neutrophil migration to the joint (Figure 5). Therefore, the potency of different CXCL9 peptides to compete with chemokines for GAG binding was investigated in more detail. Since after injection of MSU crystals in the knee cavity the presence of the murine CXCR2 ligand CXCL1 is important for neutrophil migration (49), competition of CXCL9 peptides with CXCL1 for binding to HS was evaluated. Figure 6A demonstrates that CXCL9(74-93) is a less potent competitor than CXCL9(74-103) for CXCL1 binding to HS. A threefold molar excess of CXCL9(74-103) compared to CXCL1 results in 43% reduction of CXCL1 binding to HS, whereas a 10-fold molar excess of CXCL9(74-93) only results in 39% reduction. In contrast, CXCL9(86-103) was not able to inhibit the interaction between CXCL1 and HS. Since CS is generally found in connective tissues, such as cartilage, skin, tendons, and ligaments (53, 54), the ability of CXCL9 peptides to compete with CXCL1 for binding to CS was evaluated also. Again, three times more CXCL9(74-93) than CXCL9(74-103) was needed to achieve the same reduction of CXCL1 binding to CS (Figure 6B). In addition, the maximum inhibition of CXCL1 binding achieved by competition with CXCL9(74-103) (72% for HS and 63% for CS) was higher than the maximum inhibition achieved by competition with CXCL9(74–93) (52% for HS and CS).

### CXCL9(74-103) Binds to Endothelial Cells and Prevents Adhesion of Peripheral Mononuclear Cells in the Murine Cremaster Muscle Model

**Figure 7:**
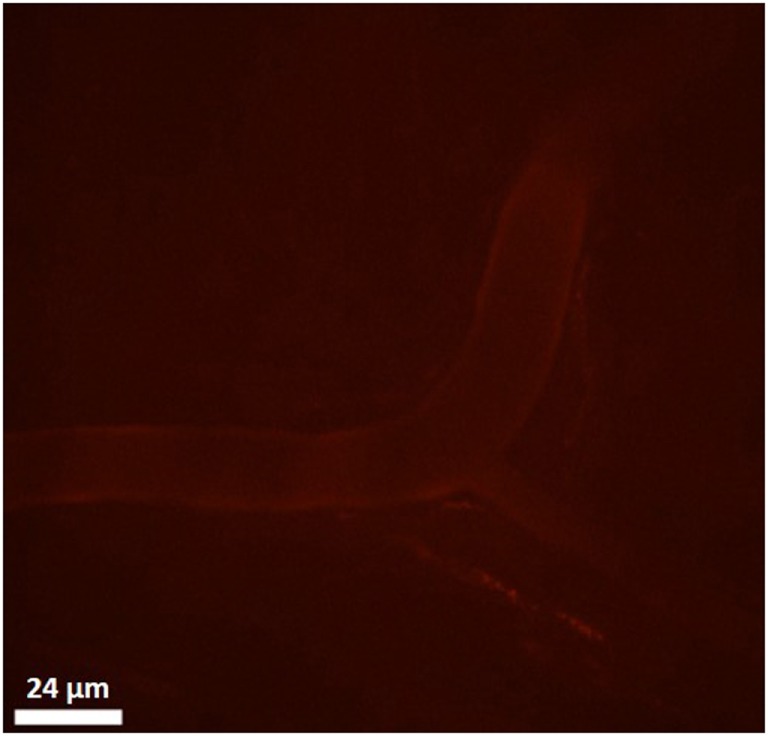
**Binding of TAMRA-labeled CXCL9(74-103) to the vascular surface of the endothelium**. Binding of the TAMRA-labeled CXCL9(74-103) to endothelial cells was evaluated in the murine cremaster muscle model. Fluorescent peptide (100 µg) was injected intravenously and binding to the vascular surface of the endothelium was visualized by confocal *in vivo* imaging 50 min after injection.

**Figure 8:**
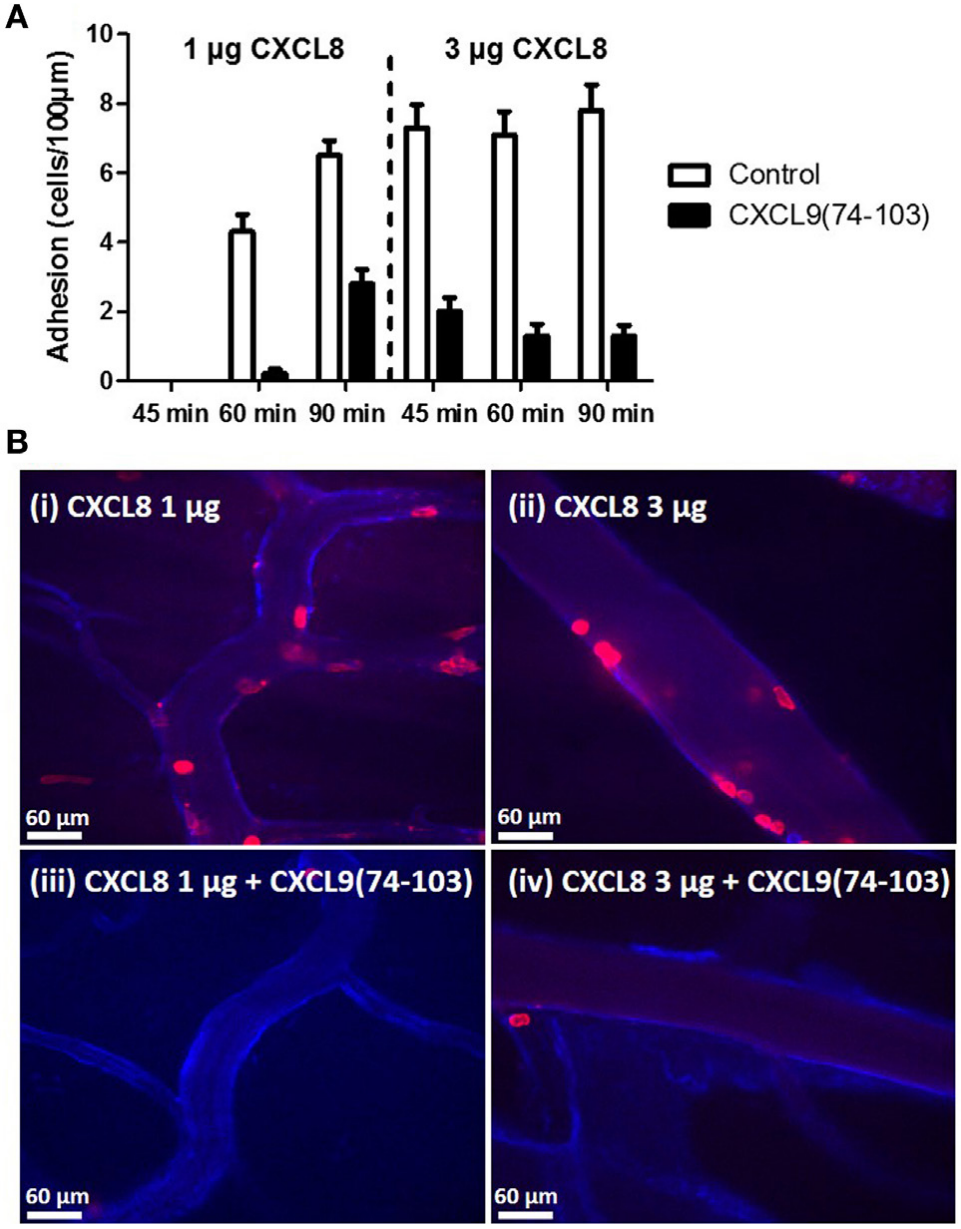
**CXCL9(74-103) inhibits adhesion of neutrophils in the murine cremaster muscle model**. **(A)** Neutrophil adhesion to the endothelium in response to an intrascrotal injection of 1 or 3 µg CXCL8 in the presence or absence of 108 µg intravenously injected CXCL9(74-103) was quantified at different time points (45, 60, and 90 min). **(B)** The cremaster of mice was superfused with 1 (i and iii) or 3 µg (ii and iv) of CXCL8 and endothelium was stained with anti-PECAM-1/CD31 antibody coupled to Alexa 647. The adhering neutrophils were visualized with an anti-Ly6G antibody conjugated to PE and quantified at 60 min post CXCL8 injection. In addition, mice were injected intravenously with either saline (i and ii) or CXCL9(74-103) (iii and iv) 5 min prior to the intrascrotal injection of CXCL8.

To evaluate the binding of CXCL9(74-103) to the endothelium *in vivo*, the peptide was site-specifically fluorescently (TAMRA-)labeled at the NH_2_-terminus avoiding modification of lysine side chains which are known to be important for interaction with GAGs. Binding of TAMRA-CXCL9(74-103) to endothelial cells was evaluated in the murine cremaster muscle model. Fifty minutes after intravenous injection, the peptide was clearly present on the vascular surface of the endothelium (Figure 7). In circulation, only a fraction of the injected amount (3% or 2 µg/ml) of the TAMRA-labeled peptide was detected in the plasma 5 min after intravenous injection (assuming a blood volume of 3 ml for the mice). Already 1 h after injection, no circulating peptide was detected in the plasma (<240 ng/ml). In addition, the effect of prior CXCL9(74–103) injection via the jugular vein on chemokine superperfusion of the cremaster muscle was analyzed. At different time intervals (45, 60, and 90 min) after CXCL8 addition to the cremaster muscle superperfusate, neutrophil-endothelial cell interactions were registered through intravital microscopy and the number of adherent neutrophils was quantified (Figure 8A). Intrascrotal injection of 1 µg CXCL8 resulted in an increase in adherent neutrophils after 60 min, whereas injection of 3 µg CXCL8 already resulted in an increase after 45 min. In both cases (1 and 3 µg CXCL8), concomitant intravenous injection of 108 µg CXCL9(74-103) reduced the adherence of neutrophils to the endothelium significantly (Figure 8B; Video S1 in Supplementary Material).

## Discussion

Directional leukocyte migration to specific primary, secondary, or tertiary lymphoid organs is important for normal functioning of our immune system. In addition, it is a crucial phenomenon to clear pathogens from sites of infection, to kill malignant cells, and to restore tissue damage. The interplay between adhesion molecules such as selectins and integrins, chemoattractants, and their receptors guides this process (17, 18, 55, 56). In order to prevent wash-out of chemokines from the surface of endothelial cells, chemokines can bind to negatively charged GAGs (18, 57, 58). These GAGs are linear polysaccharides which consist of repeating disaccharide subunits and dependent on the subunit they will be classified into different classes, e.g., heparin, HS, and CS (59). Heparin consists of repeating glucuronic acid and N-acetyl glucosamine residues and is highly sulfated with 80–90% N-sulfated glucosamine units. In contrast, HS consists of iduronic acid and is less sulfated with only 30–60% of the glucosamine units being N-sulfated. An extra level of complexity is the high degree of variations in GAG (e.g., HS) structures in different organs (60). It has been shown that chemokines localize within capillary venules in a GAG-dependent way to create a chemotactic gradient and destruction of these gradients results in abrogated directional migration of leukocytes (12, 13). Through interaction with GAGs chemokines can also form gradients in tissues along which leukocytes can migrate to the site of inflammation (13, 14). This interaction occurs between GAG-binding motifs in the chemokine and the sulfated domains in the GAG.

Three human chemokines, i.e., CXCL9, CXCL12γ, and CCL21, possess an elongated COOH-terminal tail which is highly positively charged. Linking the COOH-terminal tail of CCL21 to the related CCR7 ligand CCL19 enhanced its affinity for heparin (61). Cooperativity between CCL21 and other chemokines was shown to depend on this GAG-binding COOH-terminal domain (62) and the COOH-terminal tail of CCL21 reduced its *in vitro* chemotactic potency in a 3D dendritic cell chemotaxis assay, but enhanced its efficiency to activate ERK1/2 signaling and β-arrestin recruitment (63). In addition, full-length CCL21 was shown to induce integrin-dependent dendritic cell spreading, polarization and haptotactic movement, whereas CCL21 missing the positively charged C-terminus induced non-adhesive and integrin-independent directional migration (64). Also CXCL12γ appeared to be a weaker chemoattractant than CXCL12α in assays studying migration through uncoated chemotaxis membranes (65). However, CXCL12γ attracted T cells through an endothelial layer. The highly charged COOH-terminal domain of CCL12γ was shown to interact with sulfotyrosines on CXCR4 thereby enhancing its affinity for the receptor but reducing the signal. HS was able to inhibit binding of CXCL12γ to sulfotyrosines of CXCR4 and converted CXCL12γ into a functional CXCR4 ligand. Recently, we identified natural forms of CXCL9 missing the COOH-terminal domain that retained CXCR3 signaling potency (42).

In several inflammatory diseases, an enhanced expression of chemokines leads to the pathological and continuous accumulation of activated leukocytes in tissues and consequently tissue damage. Thus, a reduction of chemokine activity at sites of excessive inflammation is needed. Although a lot of efforts were invested in the development of small molecule chemokine receptor antagonists, since the discovery of chemokine receptors as members of the GPCR family about two decades ago, success stories are limited to a few drugs that entered the market (66). Recently, inhibition of chemokine–GAG interaction has been explored as a complementary alternative for chemokine receptor antagonists. Both modified chemokines with enhanced GAG interaction and reduced activity on GPCRs, and aptamers that interfere with chemokine–GAG binding have entered clinical development (40, 41). The COOH-terminal CXCL9 domain CXCL9(74-103) potently bound GAGs, competed for GAG binding with several chemokines and was able to inhibit CXCL8- and MSU crystal-induced leukocyte migration to joints (42). In addition, the CXCL9(74-103) and COOH-terminal CXCL12γ peptides were able to inhibit viral infection of cells with dengue virus, herpes simplex virus-1 and respiratory syncytial virus due to competition with the viruses for GAG binding (43).

In this study, we investigated in detail the role of the COOH-terminal part of CXCL9 for its affinity and specificity toward different GAGs. IFT showed that both COOH-terminal peptides, i.e., CXCL9(74-103) and CXCL9(74-93), show high and comparable affinity for LMWH and HS. CXCL9(74-93), which contains the two NH_2_-terminal and crucial GAG-binding motifs of CXCL9(74-103), competed equally efficient with CXCL8 for binding to immobilized heparin and HS. However, the affinity for CS and the binding of CXCL9(74-93) to cellular GAGs on CHO cells is lower compared to CXCL9(74-103). This suggests that on the surface of CHO cells not only HS, but a mixture of different GAGs are expressed and are important for the binding of the COOH-terminal peptides. Analogously, CXCL9(74-93) is not able to compete with CXCL8 for binding to cellular GAGs to the same degree as CXCL9(74-103). Thus, the removal of the 10 COOH-terminal amino acids of CXCL9(74-103) results in a certain specificity toward LMWH and HS (and loss of CS binding). Previously, it has been described that the motifs BBXB and BBBXXB in proteins, in which B represents a basic and X represents any non-basic amino acid, are important for GAG binding (67). Both CXCL9(74-103) and CXCL9(74-93) contain the same GAG-binding motifs (Lys^75^-Lys^78^ and Lys^85^-Lys^90^). However, CXCL9(74-93) lacks the remaining COOH-terminal amino acids. This modification influenced the interaction with GAGs, thereby indicating a certain degree of specificity of the peptides for different GAGs. It can be suggested that the change of GAG affinity and specificity between both peptides is due to the presence of a “reversed” GAG-binding domain (BXBB instead of BBXB) in the COOH-terminal region of CXCL9(74-103) between Arg-98 and Lys-101.

**Figure 9:**
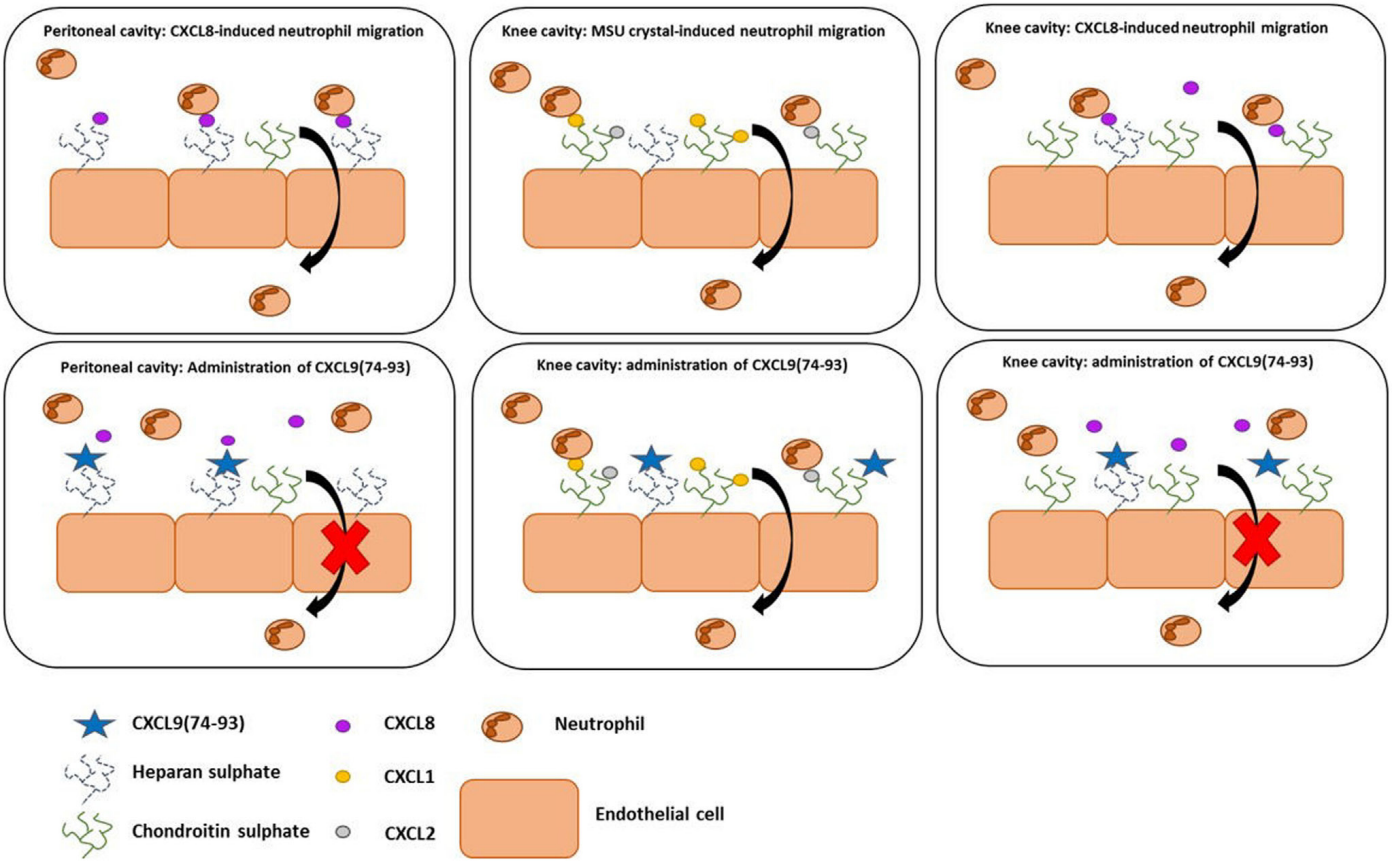
**The anti-inflammatory activity of CXCL9(74-93) in murine models of neutrophil migration**. In the peritoneum, both CXCL9(74-93) and CXCL9(74-103) inhibit CXCL8-induced neutrophil migration. Because of the high affinity of both peptides for heparan sulfate (HS), it can be suggested that HS is important for the creation of a CXCL8-dependent chemotactic gradient in the peritoneum. In that way, the CXCL9 peptides compete with CXCL8 and inhibit neutrophil extravasation. Although chondroitin sulfate (CS) is most abundant in the articular cavity and the affinity of the peptides for CS is different, both peptides are able to inhibit CXCL8-induced neutrophil migration to the knee cavity. In the gout model, the lower efficiency of CXCL9(74-93) to inhibit CS binding of the induced murine chemokines does not prevent neutrophil migration.

In view of the variation in GAG structures between different organs, we investigated the importance of chemokine-GAG interactions in the inflammatory response upon injection of CXCL8 or MSU crystals in different tissues, i.e., the peritoneal and/or knee cavity and the cremaster muscle. Although mice lack the gene for CXCL8, a strong neutrophilic inflammation can be induced when CXCL8 is administered in mice (42, 68). In addition, the capacity of CXCL9(74-93) to inhibit MSU crystal-induced neutrophil migration was assessed. Both upon intraperitoneal or intra-articular injection of CXCL8, concomitant injection of CXCL9(74-103) or CXCL9(74–93) was able to inhibit neutrophil-dependent inflammation. Our results also show that CXCL9(74-93), when given intravenously in equimolar concentrations, could inhibit CXCL8 activity *in vivo* with similar potency as CXCL9(74-103). On the contrary, CXCL9(74-93) was not able to inhibit neutrophil extravasation in the more complex and less chemoattractant-specific MSU crystal-induced gout model. This difference in anti-inflammatory activity can be explained in several ways (Figure 9). First, it is known that in the articular cavity CS is an abundant GAG, comprising 60–90% of the GAG molecules (69, 70). Keeping in mind that CXCL9(74-93) binds with lower affinity to CS, it can be suggested that this peptide cannot compete with chemokines for binding to CS in the same way as CXCL9(74-103). Indeed, CXCL9(74-93) is a less potent competitor for CXCL1 binding to HS and CS (Figure 6), a previously reported major neutrophil chemoattractant in this gout model (49). Second, it is known that chemokines bind with different affinity and/or specificity to different GAGs (71, 72). In the gout model, the induced murine chemokines (CXCL1 and CXCL2 (49)) probably bind with different affinity or specificity to different GAGs in the murine knee cavity than human CXCL8. This would explain why a peptide with lower affinity for those GAGs cannot compete with the induced murine chemokines. In conclusion, both the affinity and the specificity of the peptides and the chemokines for different GAGs will determine whether competition can occur. Finally, we investigated whether the more potent CXCL9(74-103) peptide could inhibit the activity of CXCL8 in the murine cremaster muscle model. In this model, CXCL8 was injected intrascrotally into the cremaster muscle to induce adhesion of neutrophils to the endothelium. Intravital microscopy of the muscle clearly shows that the fluorescent CXCL9 peptide binds to the endothelium. In this way, CXCL8 was not able to bind to GAGs and direct evidence was provided for lower adhesion of neutrophils to the endothelium.

Thus, overall we show that CXCL9(74-103) indeed inhibits neutrophil migration *in vivo* through interference with GAG interactions in three animal models and that shortening this CXCL9 from the COOH-terminus increases its specificity for particular GAGs. Obtaining detailed knowledge on the specificity of these GAG-binding peptides is important for the development of lead molecules for the generation of therapeutics competing with active chemokines for GAG binding to avoid potential side effects.

## Ethics Statement

Animal experiments at the Rega Institute for Medical Research, University of Leuven, were carried out in female adult NMRI mice, purchased from Elevage Janvier (Le Genest Saint Isle, France), in agreement with the Ethical Committee for Animal Care and Use of the KU Leuven and in adherence to the international guidelines for animal ethics and welfare. Mouse studies at the Federal University of Minas Gerais were performed in adult C57BL/6 mice. The experiments were approved by the Animal Ethical Committee of the Federal University of Minas Gerais. Mice were sacrificed by i.p. injection of an overdose of nembutal or ketamine (150 mg/kg) and xylazine (20 mg/kg) prior to the collection of joint lavages. For the intravital microscopy studies, male C57Bl/6J mice were purchased from Jackson Laboratories (Bar Harbor, ME, USA). Animals were maintained in a specific pathogen-free environment at the University of Calgary Animal Resource Centre. All experimental animal protocols were approved by the University of Calgary Animal Care Committee and were in compliance with the Canadian Council for Animal Care Guidelines.

## Author Contributions

VV, AM, EK, SS, and PP designed the study and VV, EK, SS, and PP wrote the paper. All authors analyzed and interpreted the data. VV, DB, RJ, BP, and NB performed the experiments. VV and PP synthetized and purified the CXCL9 peptides and performed mass spectrometry and GAG-binding assays. VV, AM, and NB were involved in the CXCL8-induced inflammation model in the peritoneum. BP, EK, and PK were involved in the cremaster muscle model. DB, RJ, FA, and MT were involved in the murine gout model. VV and AK were involved in the IFT measurements. All authors revised the work and approved the version to be published.

## Conflict of Interest Statement

The authors declare that the research was conducted in the absence of any commercial or financial relationships that could be construed as a potential conflict of interest.

## Supplementary Material

Video S1 **Adhesion of neutrophils in the murine cremaster muscle model is inhibited by a single injection of CXCL9(74-103)**. Adhesion of CXCL8 activated neutrophils to endothelium of the cremaster muscle vasculature. Mice were injected intravenously with either saline (controls; video I) or CXCL9(74-103) (video II) 5 min prior to an intrascrotal injection of 1 µg of CXCL8. The adhering neutrophils were visualized (as shown in exemplary videos I and II) and quantified (Figure 7B) at 60 min post CXCL8 injection. The endothelium was stained with anti-PECAM-1/CD31 antibody coupled to Alexa 647 and neutrophils with anti-Ly6G antibody conjugated to PE. The scale bar indicates 60 µm.
